# Ethanolic extract of propolis inhibits atherosclerosis in ApoE-knockout mice

**DOI:** 10.1186/1476-511X-12-123

**Published:** 2013-08-13

**Authors:** Yongqi Fang, Hui Sang, Na Yuan, Hongli Sun, Shutong Yao, Jiafu Wang, Shucun Qin

**Affiliations:** 1 College of Basic Medical Sciences, Taishan Medical University, Taian 271000, China; 2 Institute of Atherosclerosis, Key Laboratory of Atherosclerosis in Universities of Shandong, Taishan Medical University, Taian 271000, China; 3 School Hospital, Taishan Medical University, Taian 271000, China

**Keywords:** ApoE-knockout mice, Atherosclerotic lesion, Ethanolic extract of propolis

## Abstract

**Background:**

The present study was undertaken to investigate the effects and underlying mechanism of ethanolic extract of propolis (EEP) on the development of atherosclerotic lesions in ApoE^−/−^ mice.

**Methods:**

Eight-week-old male ApoE^−/−^ mice fed a high-fat diet were treated with EEP (160 mg/kg/d) or vehicle (the same dose) respectively for 14 weeks. The serum levels of total cholesterol (TC), high-density lipoprotein cholesterol (HDL-C) and triglycerides (TG) were determined by enzymatic methods. Non-HDL-C was calculated as TC minus HDL-C. Serum interleukin-6 (IL-6), interleukin-17 (IL-17), endothelin (ET), inducible nitric oxide synthase (iNOS) and vascular endothelial growth factor (VEGF) were determined with enzyme-linked immunosorbent assay methods. Nitric oxide (NO) content was measured with an enzymatic nitrate reductase assay. Analyses of atherosclerotic lesions in whole aorta and aortic root sections were performed with plaque staining using Oil Red O.

**Results:**

Compared with the vehicle-treated group, serum contents of total cholesterol (TC), triglycerides (TG) and non-HDL-C reduced significantly by 31.88%, 21.01%, and 27.11% respectively in the EEP-treated group. Administration of EEP decreased the level of IL-6 and increased the level of IL-17 in ApoE^−/−^ mice with a high-fat diet. Compared with the vehicle-treated group,EEP significantly reduced the levels of ET and VEGF,and showed a trend to increase NO and inhibit iNOS. In the ApoE^−/−^ mice fed a high-fat diet, EEP significantly reduced atherosclerotic lesion development in the aortic root and whole aorta.

**Conclusion:**

EEP can inhibit atherosclerotic lesion formation in ApoE^−/−^ mice fed a high-fat diet possibly through modulating cholesterol, regulating inflammatory reaction,inhibiting ET and VEGF, and protecting vascular endothelial cells.

## Background

Atherosclerotic disease is the main cause of death in humans and presents long-term challenges for those working in medical science. Sufficient evidence indicates that inflammation plays an important role in the genesis, development and progression of atherosclerosis (AS) [1,2]. The damage or dysfunction of vascular endothelial cells induced by pro-inflammatory factors, especially dyslipidemia, is the starting and key point of AS. Many clinical studies have demonstrated that impaired NO-dependent vasodilatation is closely related to atherosclerosis [3]. Hypercholesteremia is an independent and significant risk factor of atherosclerosis. Lipid-lowering interventions are the cornerstone for the prevention and treatment of atherosclerosis. Thus, inhibiting inflammatory reaction and improving the function of vascular endothelial cells is a vital strategy to combat atherosclerotic lesion progression.

Propolis is a sticky substance made from plant saps and resins, collected by honey bees and mixed by them with wax flakes and pollen. Their salivary secretions join with these compounds to create a unique substance with a very complex chemical composition. The active ingredients are flavanoids. Previous studies show that propolis possesses anti-inflammatory, antioxidant, immunomodulatory, anti- proliferative, antibacterial, and antiviral properties [4-7]. Koya-Miyata S et al. reported that propolis prevents diet-induced hyperlipidemia and mitigates weight gain in diet-induced obesity in C57BL/6 N mice [8]. The study by Nader MA et al., suggests that ethanolic extract of propolis (EEP) produced protective effects on the development of atherosclerosis in cholesterol-fed rabbits [9]. These studies indicate that propolis may reduce the risk of AS and have the potential of being anti-AS, and we believe this evidence should be sustained through more experiments in vivo. Therefore we observed the effects of EEP on atherosclerotic plaque and explored the underlying mechanism with ApoE^−/−^ mice.

## Materials and methods

### Animals

16 eight-week-old male ApoE^−/−^ mice were supplied by the Laboratory Animal Center of Peking University, weight 22 ± 2 g. The mice were kept in a temperature- and humidity-controlled room with a 12/12 h light–dark cycle. All experiments were approved by the Laboratory Animals’ Ethical Committee of Taishan Medical University and followed national guidelines for the care and use of animals. All ApoE^−/−^ mice were fed a Western-type diet (21% fat and 0.15% cholesterol) and randomly divided into 2 groups (n=8), the EEP group (treated with EEP, 160 mg/kg/d) and the control, or model group (treated with vehicle, same dose). Both EEP and vehicle were administered intragastrically once daily for 14 weeks, with dose adjustment weekly according to body weight.

### Main reagents

The crude propolis was obtained from the Taishan bee yard in China. EEP was extracted as described previously [8]. Interleukin-6 (IL-6), interleukin-17 (IL-17), endothelin (ET) and vascular endothelial growth factor (VEGF) enzyme-linked immunosorbent assay (ELISA) reagent kits were purchased from R&D Systems Inc (Minnesota, USA), Nitric oxide (NO) and inducible nitric oxide synthase (iNOS) reagent kits were supplied by Nan-jing Jiancheng Bioengineering Institute (Nanjing, China). Oil Red O was obtained from Sigma-Aldrich Co. St. Louis (USA).

### Serum analysis

After 14 weeks of treatment, blood was collected from the retro-orbital sinus of all mice after a 12 h fasting period. The serum levels of total cholesterol (TC), high density lipoprotein cholesterol (HDL-C), and triglycerides (TG) were determined by enzymatic methods. Non-HDL-C was calculated as TC minus HDL-C. The serum levels of IL-6, IL-17, ET, VEGF and iNOS were determined by ELISA kits. The serum content of NO was measured by nitrate reductase method.

### Lesion analysis

Following 14 weeks of treatment, the mice were anesthetized with an intraperitoneal injection of sodium pentobarbital (40 mg/kg) and blood was collected. The mice were sacrificed by exsanguination and perfused with 10 ml ice-cold phosphate-buffered solution (PBS) at physiological pressure via the left ventricle. The aortas were isolated and the adventitia was thoroughly cleaned under a dissecting microscope, they were then cut open longitudinally and stained with Oil Red O. The percentage of the plaque area stained by Oil Red O to the total luminal surface area was determined.

To quantify the atherosclerotic lesions in the aortic root, serial cryostat sections (8 μm) were prepared as described previously [10]. In brief, atherosclerotic lesions in the aortic root were examined at 3 locations and each separated by 120 μm, 4 to 5 serial sections were prepared from each location. The sections were stained with Oil Red O and the lipid composition of the lesion was determined by calculating the percent of the Oil Red O positive area to the total cross-sectional vessel wall area. All images were captured with an Olympus BX51 microscope equipped with a video camera and analyzed using Image–Pro-Plus 6.0 software (version 6.0, Media Cybernetics, MD, USA). In each case, the average value for 4 to 5 locations or sections of each animal was used for analysis.

### Statistical analysis

Statistical analysis was performed by t-tests. Data were expressed as means ± standard deviations (SD). *P* values less than 0.05 were considered statistically significant.

## Results

### EEP decreased atherosclerotic lesions in the aorta of ApoE^−/−^ mice

**Figure 1:**
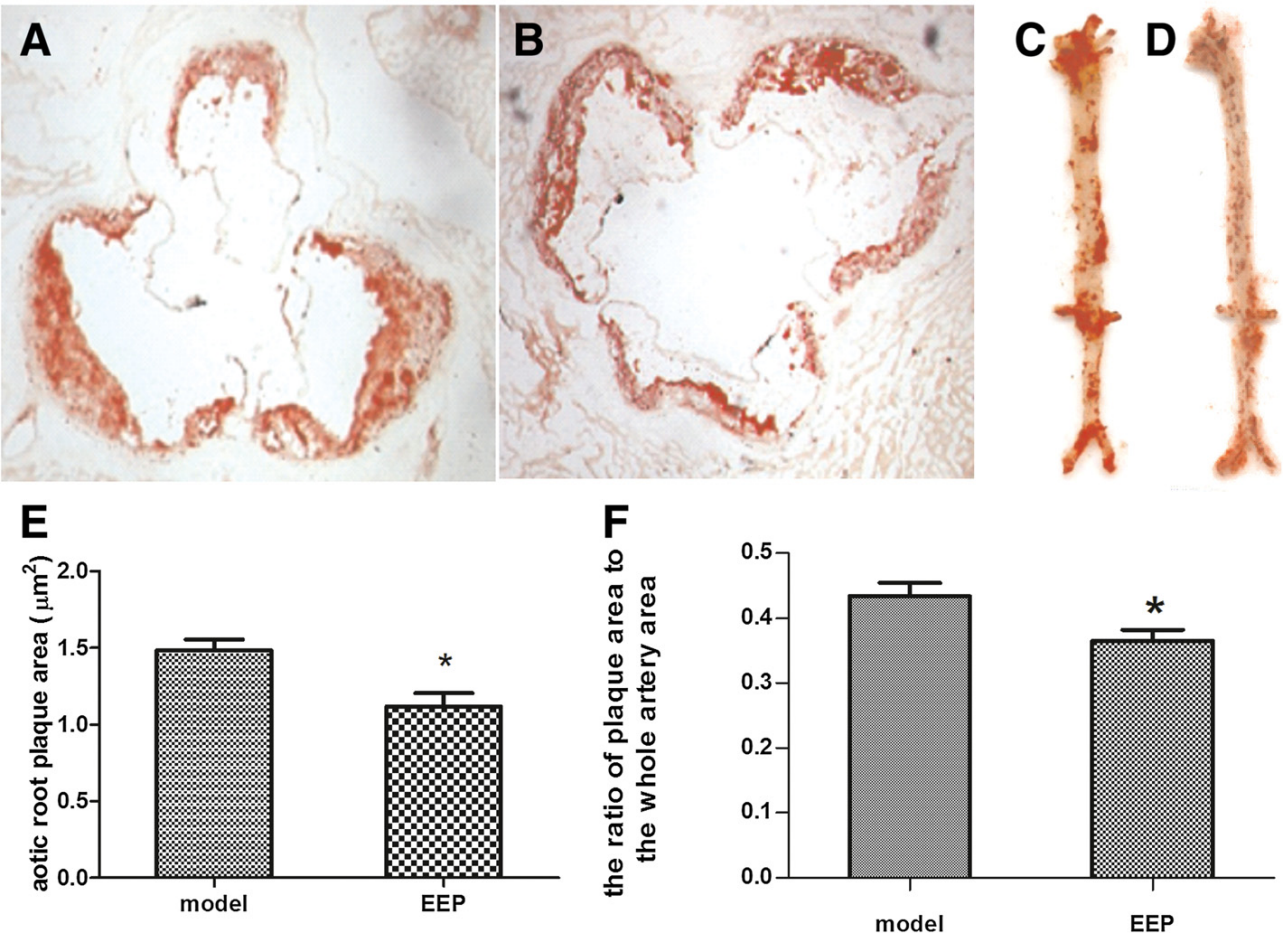
**EEP reduced atherosclerotic lesions in the aorta of ApoE**^**−/− **^**mice fed a high-fat diet. A**, **B** Representative of Oil Red O-stained aortic cross-sections. Orange-red area is lipid plaque, 10×4 magnification. **A**, model group; **B**, EEP group. **C**, **D** Representative of Oil Red O-stained the full length of the aorta. Orange-red area is lipid plaque, 10×4 magnification. **C**, model group; **D**, EEP group. **E**. Quantitation of lesion areas in Oil Red O-stained aortic cross-sections by Image-Pro Plus software. **F**, Quantitation of atherosclerotic lesions in the full length of the aorta by Image-Pro Plus software. ^*^P<0.05 versus model group. EEP, ethanolic extract of propolis.

As shown in Figure 1, the orange-red area exhibits lipid deposition in the aorta. In the EEP group, formation of atherosclerotic lesions in cross-sections of the aortic valve area decreased significantly by 25.26% compared with the model group (Figures 1A, 1B, 1E). Oil Red O staining revealed atherosclerotic plaque in the full length of the aorta, distributed mainly in the aortic arch and the areas surrounding the branching points of the major arteries. EEP also reduced the aortic lesion area (lesion area compared to total aortic area) by 16.17% compared with the model group (Figures 1C, 1D, 1F).

### Effects of EEP on serum lipid in ApoE^−/−^ mice

**Figure 2:**
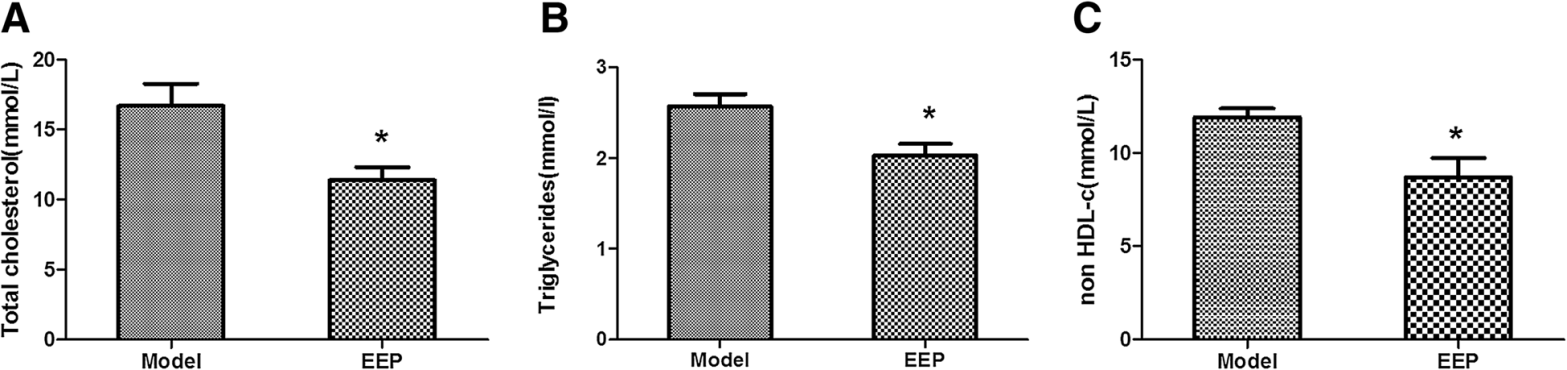
**Effects of EEP on serum lipid in ApoE**^**−/− **^**mice fed a high-fat diet.** Serum lipid concentrations after a 14-week supplementation period with EEP (160 mg/kg/d) in ApoE^−/−^ mice on a high-fat diet. **A**, total cholesterol (mmol/L); **B**, triglycerides (mmol/L); **C**, non-HDL-C (mmol/L). Data are presented as means ± SD (n=8 per group). ^*^P<0.05 versus model group. EEP, ethanolic extract of propolis; HDL-C, high-density lipoprotein cholesterol.

Figure 2 shows hyperlipidemia in the ApoE^−/−^ mice, induced by a high-fat diet for 14 weeks, and that serum levels of TC, TG and non-HDL-C were significantly reduced by 31.88%, 21.01%, and 27.11% respectively in the EEP group, compared with the model group. However, EEP had no significant effect on serum HDL-C compared with the model group (the data was not shown).

### Effects of EEP on serum ET, NO, iNOS, VEGF, IL-6 and IL-17 in ApoE^−/−^ mice fed a high-fat diet

**Figure 3:**
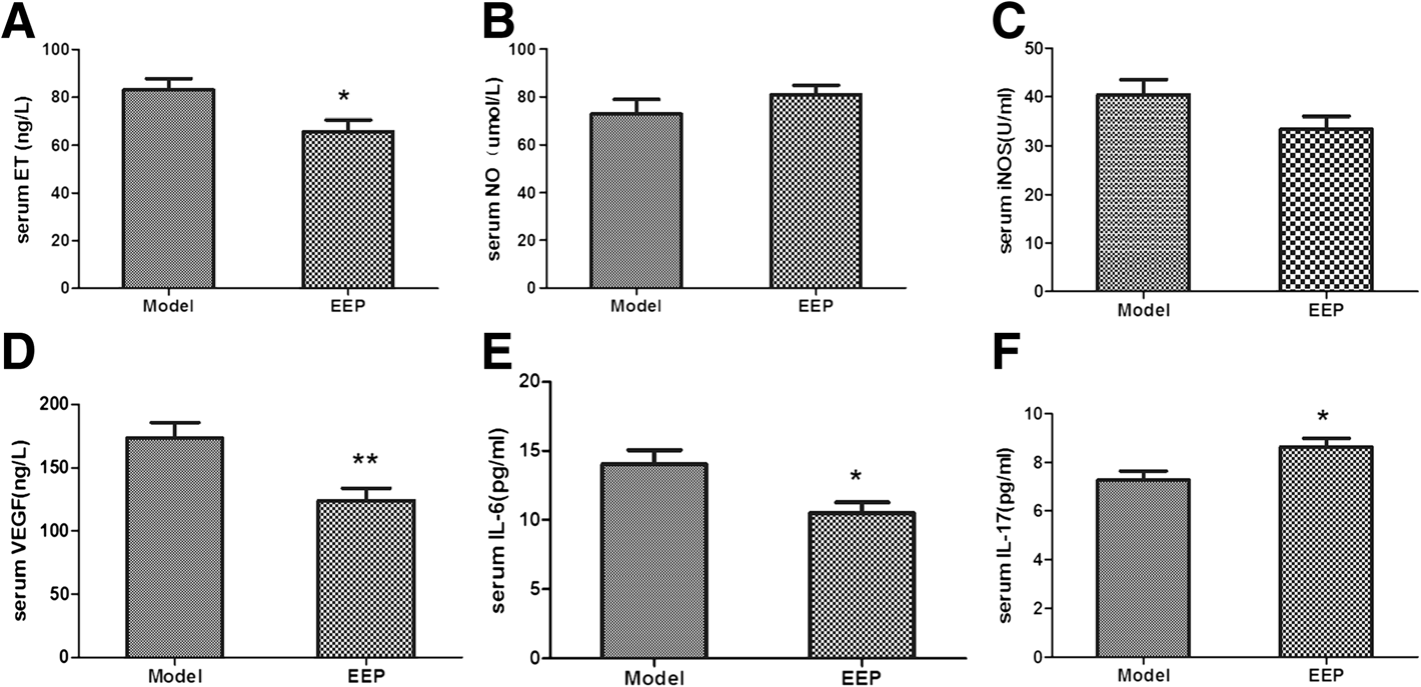
**Effects of EEP on serum ET, NO, iNOS, VEGF, IL-6 and IL-17 in ApoE**^**−/− **^**mice fed a high-fat diet.** Serum concentrations of ET, NO, iNOS, VEGF, IL-6 and IL-17 after a 14-week supplementation period with EEP (160 mg/kg/d) in ApoE^−/−^ mice on a high-fat diet. **A**, ET (ng/L); **B**, NO (umol/L); **C**, iNOS (U/mL); **D**, VEGF (ng/L); **E**, IL-6 (pg/ml); **F**, IL-17 (pg/ml). Data are presented as means ± SD (n=8 per group). ^*^P<0.05; ^**^P<0.01 versus model group. EEP, ethanolic extract of propolis; ET, endothelin; NO, nitric oxide; iNOS, inducible nitric oxide synthase; VEGF, vascular endothelial growth factor; IL-6, interleukin-6; IL-17, interleukin-17.

As shown in Figures 3A, 3B, 3C, the serum concentrations of ET decreased significantly by 20.96% in the EEP group compared with the model group (65.72 versus 83.15 ng/L). Supplementation with EEP (160 mg/kg/d) increased NO and decreased iNOS, yet no statistical significance was observed.

In the present study, serum concentration of VEGF expression was detected by ELISA. As shown in Figure 3D, supplementation with EEP (160 mg/kg/d) in ApoE^−/−^ mice fed a high-fat diet significantly inhibited VEGF by 28.45% compared with the model group (124.05 versus 173.37 ng/L).

As shown in Figures 3E, 3F, supplementation with EEP (160 mg/kg/d) in ApoE^−/−^ mice fed a high-fat diet significantly decreased the level of IL-6 by 25.30% (10.48 versus 14.03 pg/ml) and increased the level of IL-17 by 18.77% (8.64 versus 7.27 pg/ml), compared with the model group.

## Discussions

A number of studies have demonstrated that propolis may possibly inhibit atherosclerosis. Fuliang Hu et al. [11] and Nader MA et al. [9] respectively confirmed with rat and rabbit models that propolis can reduce atherosclerotic lesion formation. In the present study,we used ApoE^−/−^ mice to observe the effects of EEP on atherosclerotic lesions and to explore the underlying mechanism. The ApoE^−/−^ mouse, a recognized atherosclerosis animal model, can spontaneously develop atherosclerosis with features similar to those observed in human hyperlipoproteinemia. These lesions may develop even with a normal diet, while a high fat diet can accelerate atherosclerotic lesion formation [12]. Our present study has shown that EEP can inhibit atherosclerotic lesion formation in whole aorta and aortic root sections in ApoE^−/−^ mice fed a high-fat diet. The possible mechanisms may be mediated by modulating cholesterol, regulating inflammatory reactions, inhibiting ET and VEGF expression, and protecting vascular endothelial cells.

Hypercholesterolemia is recognized as a risk factor for atherosclerosis. Propolis may possibly protect against atherosclerosis by regulating cholesterol. Fuliang Hu et al. [11] and Nader MA et al., [9] reported on Sprague Dawley rats and New Zealand rabbit models respectively, that propolis can decrease TC, TG and LDL-C. Koya-Miyata S et al. reported that propolis prevents diet-induced hyperlipidemia and mitigates weight gain in diet-induced obesity in C57BL/6 N mice [8]. Our results showed that EEP significantly inhibited TC, TG and non-HDL-C in ApoE^−/−^ mice fed a high-fat diet. The fact that EEP can significantly inhibit AS suggests that the modulation of lipid metabolism contributes to the anti-atherogenic effect of EEP.

Atherosclerosis is considered a chronic inflammatory disease [1]. Inflammatory reactions exist throughout the atherosclerosis process. IL-6, an inflammation pre-stimulation monokine secreted by mononuclear-macrophages with moderate inflammatory activity, is strongly associated with atherosclerosis and its progression [13]. In rats with Freund’s complete adjuvant induced arthritis, propolis extracts significantly inhibited the increase of IL-6 in inflammatory tissues [5]. Recently, a new lineage of CD4^+^ T cells, type 17 helper T cells producing the signature cytokines IL-17, IL-21, and IL-22, has been identified [14]. The relevance of IL-17 to human atherosclerosis remains poorly defined because of conflicting results in animal studies [15]. An up-to-date study by Simon T et al. [16] found that elevated levels of IL-17 are associated with a better outcome in patients with acute myocardial infarction, supporting a protective regulatory role of IL-17 in coronary heart disease,whereas IL-6 levels were associated with a worse outcome despite the fact that IL-6 and IL-17 might induce each other. Our study showed that administration with EEP significantly decreased the level of IL-6 and increased the level of IL-17 in ApoE^−/−^ mice on a high-fat diet. Therefore in the early stages of atherosclerosis,inhibition of IL-6 and elevation of IL-17 by EEP might be one mechanism of its anti-AS effects.

The damage or dysfunction of vascular endothelial cells, induced by various kinds of proinflammatory factors especially dyslipidemia,is the initial and key point of atherosclerosis. The disequilibrium of endothelin (ET) and nitric oxide (NO) is the main cause of endothelial function disorder. ET-1, a 21-amino acid peptide with strong vasoconstrictor and mitogenic properties [17], is the predominant molecular form of endothelium involved in the formation of artherosclerosis. NO might inhibit all key processes participating in the early pathogenesis. Active iNOS can be found at early stages of atherosclerotic plaque, which leads to nitration of NO and finally aggravates AS [18]. Our results showed that EEP can significantly reduce the levels of serum ET, and meanwhile show a trend to increase NO and inhibit iNOS, although not with statistical significance. These results indicate that EEP might reverse the unfavorable imbalance between NO and ET.

Vascular endothelial growth factor (VEGF), an endothelial specific growth and chemotactic factor, can be synthesized by various cell types including macrophages, T lymphocytes and mast cells [19]. In unstable human coronary plaques, there is an increased neovascularization, which probably induced by VEGF-A [20]. Pelisek J et al. found a close association between neovascularization, expression of VEGF, inflammation, and plaque vulnerability in patients with advanced carotid stenosis [21]. Our results showed that EEP significantly reduced the levels of serum VEGF in ApoE^−/−^ mice on a high-fat diet,which indicates that EEP may reduce the quantity of developing neovascularization and thus stabilize the plaque.

## Conclusions

In conclusion, we believe that EEP can inhibit atherosclerotic lesion formation in ApoE^−/−^ mice fed a high-fat diet, possibly through modulating cholesterol, regulating inflammatory reactions,inhibiting ET and VEGF, and protecting vascular endothelial cells. More detailed mechanisms need to be investigated.

## Abbreviations

ApoE: Apolipoprotein E; ApoE−/−: Apolipoprotein E knockout; AS: Atherosclerosis; EEP: Ethanolic extract of propolis; ET: Endothelin; HDL-C: High-density lipoprotein cholesterol; IL-17: Interleukin-17; IL-6: Interleukin-6; iNOS: Inducible nitric oxide synthase; NO: Nitric oxide; TC: Total cholesterol; TG: Triglyceride; VEGF: Vascular endothelial growth factor.

## Competing interests

The authors declare that they have no competing interests.

## Authors’ contributions

YF, HS performed most of the experiments and prepared the manuscript. NY carried out the animal studies and biochemical analysis. HS carried out data collection and analysis. SY helped to draft the manuscript. JW participated in the study’s design and coordination. SQ conceived the study, participated in its design and coordination, corrected the manuscript and supervised the project. All authors read and approved the final manuscript.
